# From Sequences to Food Webs: DNA Metabarcoding Reshapes Fish Trophic Ecology

**DOI:** 10.3390/ani16030443

**Published:** 2026-01-31

**Authors:** Lin Liang, Jiajie Li, Shiyun Fang, Cheng Jiang, Sheng Bi, Lei Zhou

**Affiliations:** 1 College of Marine Sciences, South China Agricultural University, Guangzhou 510642, China; lianglin0505@stu.scau.edu.cn (L.L.); lijiajietong@foxmail.com (J.L.); fangshiyun@stu.scau.edu.cn (S.F.); 2 School of Life Sciences and Environmental Resources, Yichun University, Yichun 336000, China; jiangcheng0508@163.com; 3 University Joint Laboratory of Guangdong Province, Hong Kong and Macao Region on Marine Bioresource Conservation and Exploitation, Guangzhou 510642, China

**Keywords:** DNA metabarcoding, fish diet, food web dynamics, ecological interactions, fisheries management

## Abstract

Fish occupy pivotal trophic positions in aquatic ecosystems, mediating energy transfer and shaping community structure through their feeding interactions. Unraveling these dietary relationships is therefore fundamental for understanding ecosystem functioning and supporting sustainable fisheries management. Traditional morphological analyses, while informative, often fall short in resolving fine-scale prey diversity and trophic linkages. In contrast, DNA metabarcoding has revolutionized dietary studies by enabling comprehensive, high-resolution, and non-invasive characterization of prey assemblages. This review synthesizes recent progress in applying DNA metabarcoding to fish trophic ecology, emphasizing technical innovations, methodological standardization, and ecological insights. We discuss how DNA metabarcoding has advanced the understanding of food web complexity, species interactions, and ecological responses to environmental change. However, challenges persist in quantification accuracy, reference database completeness, and cross-source contamination. Future research integrating multi-marker approaches, standardized workflows, and multi-method integration holds promise for transforming DNA metabarcoding into a powerful, reliable and mechanistic tool for trophic ecology. Collectively, these developments will bridge molecular data with ecological theory, strengthening the scientific foundation for ecosystem-based fisheries management.

## Simple Summary

Aquatic ecosystems rely on complex feeding relationships to function properly, and fish are an important part of these relationships. Knowing what fish eat helps reveal how ecosystems respond to pollution, climate change, and biological invasions. In recent years, DNA-based methods have changed how scientists study fish diets by identifying food remains from genetic traces, even when prey cannot be seen or identified by eye. This review summarizes the use of DNA metabarcoding in fish feeding ecology and the improvements it brings to diet analysis in complex aquatic environments. We highlight how these approaches have improved understanding of feeding strategies, species coexistence, food web organization, and the ecological impacts of invasive fish. We also discuss current challenges and future directions for making DNA-based dietary studies more reliable and ecologically meaningful. Overall, DNA metabarcoding represents a promising tool for improving dietary information that supports ecosystem protection, fisheries management, and responses to environmental change.

## 1. Introduction

Species interactions play a fundamental role in regulating biodiversity and community assembly processes in aquatic ecosystems, thereby contributing to ecosystem stability [1,2]. Understanding how these interactions maintain ecosystem diversity, functionality, and evolutionary trajectories represents a major challenge in ecology [3]. Resource partitioning, an outcome of ecological interactions, defines niche boundaries and promotes the efficient use of available resources [4]. Among various ecological processes, trophic interactions, particularly predator–prey relationships, have long been central to ecological theory, as they provide key insights into species coexistence, community structure, and biodiversity conservation [4,5,6]. In aquatic environments, these complex interactions are most prominently exemplified by fish communities.

Fish play a multi-faceted and indispensable role in aquatic ecosystems, serving as both ecological cornerstones and vital socioeconomic resources [7,8]. As key consumers, they drive nutrient cycling and energy transfer, thereby maintaining ecosystem stability and resilience [9]. Moreover, fish communities serve as valuable bioindicators of aquatic ecosystem health, as changes in community composition often reflect environmental alterations and anthropogenic pressures [10]. Through their trophic interactions and habitat-modifying behaviors, fish communities regulate key ecological processes that underpin ecosystem functioning and resilience [10,11]. Beyond their ecological roles, fish also constitute an essential renewable biological resource, supporting global food supply, livelihoods, and economic development. Therefore, a comprehensive understanding of fish community structure and the mechanisms governing their trophic relationships is vital not only for biodiversity conservation but also for the sustainable management of fisheries and aquatic ecosystems.

To decipher these complex trophic relationships, investigating diet composition provides a powerful means to understand ecological niches and resource partitioning [12,13]. As apex or meso-predators, fish exert cascading effects on energy flow within aquatic ecosystems, making their feeding ecology a window into food web organization [14,15]. This knowledge is critical for elucidating trophic pathways, characterizing species interactions, and informing management strategies that sustain both ecosystem function and fishery productivity [16].

Traditional dietary analysis has relied heavily on morphological identification of prey remains. While this approach provides direct evidence of diet, it is constrained by low taxonomic resolution, high labor demands, and limitations imposed by the digestibility of food item [17]. Complementary methods, such as stable isotope analysis and fatty acid biomarkers, have been developed to overcome some of these limitations. However, stable isotope approaches require comprehensive reference baselines that can vary spatially and temporally and may not accurately reflect the trophic level of individual species [18,19,20]. Fatty acid biomarkers offer insights into nutrient sources, but their interpretation is complicated by low taxonomic specificity, metabolic modification within consumers, and environmental influences [21]. Collectively, these limitations highlight the need for more sensitive, high-throughput, and taxonomically precise approaches to dietary studies. Although DNA barcoding has improved species identification accuracy since its introduction in 2002, its reliance on low-throughput, first-generation sequencing limits applications in complex dietary analyses [22].

The advent of high-throughput sequencing has transformed dietary research through the development of DNA metabarcoding—a powerful and sensitive approach that enables large-scale identification of prey DNA from mixed samples [23,24,25]. This approach has been successfully applied across diverse taxa, including mammals, amphibians, birds, reptiles, and fish [26,27]. In fish feeding ecology, DNA metabarcoding surpasses traditional methods by eliminating the dependence on visually identifiable remains, enabling the analysis of degraded or digested material, and providing high-resolution, high-throughput insights into dietary diversity at reduced cost and effort [28,29,30]. While DNA-based techniques may involve higher initial investment in specialized equipment and reagents, they become more cost-effective than traditional methods in large-scale studies due to reduced labor hours and the ability to process samples in parallel.

**Figure 1:**
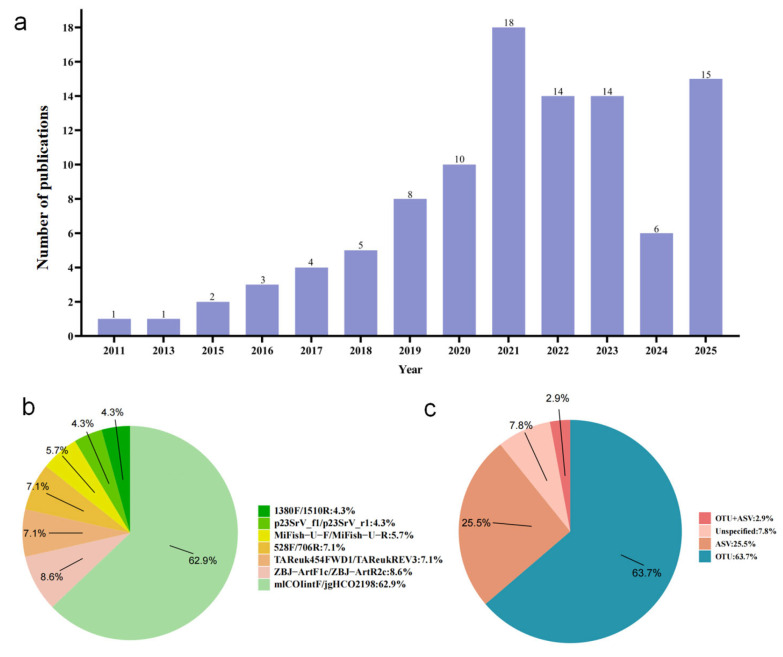
Application of DNA metabarcoding in fish diet analysis. (**a**) Annual number of publications investigating diet using DNA metabarcoding; (**b**) relative proportions of the major DNA metabarcoding primer sets used across the 101 reviewed studies; (**c**) relative proportions of bioinformatic analysis strategies employed in the 101 reviewed studies.

Given the complexity of fish trophic interactions and the ecological importance of feeding behavior, applying more sensitive and efficient tools is imperative. To evaluate the development and application of DNA metabarcoding in fish dietary research, we conducted a comprehensive literature review in October 2025 using Google Scholar, Web of Science, and China National Knowledge Infrastructure (CNKI). Searches with the keywords “DNA metabarcoding” and “fish diet” yielded 101 relevant publications from 2011 to 2025. Despite the increasing number of publications in recent years (Figure 1a), studies applying DNA metabarcoding to fish trophic ecology remain relatively scarce, highlighting that this field is still in its early developmental stage. In this context, this review synthesizes methodological advances, summarizes ecological insights gained from DNA metabarcoding studies, and discusses current challenges and future perspectives. Our aim is to provide an integrated understanding of how DNA metabarcoding is reshaping dietary research and advancing the study of fish feeding ecology.

## 2. The Experimental Design for Investigating Fish Diets Using DNA Metabarcoding Technology

**Figure 2:**
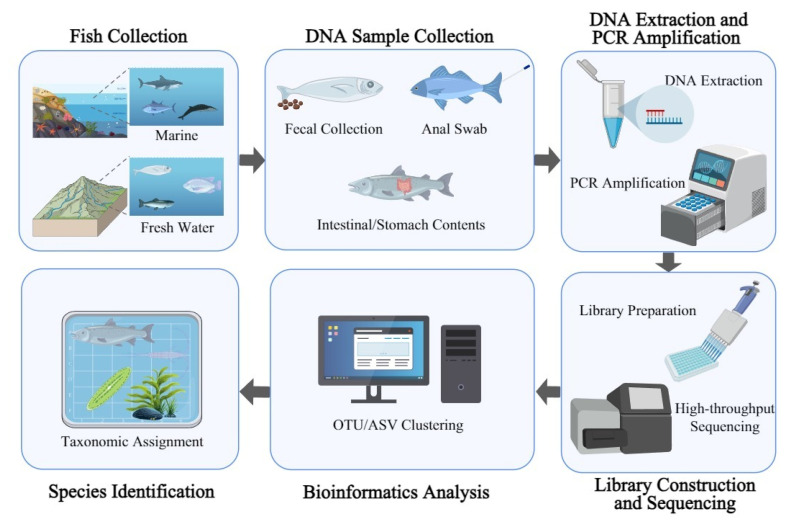
Schematic diagram illustrating the general workflow of DNA metabarcoding for fish diet analysis.

Fish diet analysis based on DNA metabarcoding generally involves the collection of gastrointestinal contents or non-invasive fecal samples, followed by DNA extraction, amplification of selected barcode regions, high-throughput sequencing, and downstream bioinformatics processing for taxonomic assignment, ultimately yielding qualitative or semi-quantitative dietary profiles (Figure 2).

DNA metabarcoding allows for species-level identification by sequencing genetic material and matching the resulting sequences to a curated reference database [31]. The accuracy and reliability of this approach are largely governed by experimental design—particularly sampling strategies, preservation methods, primer selection, reference database quality, and bioinformatics pipelines.

### 2.1. Sampling Strategies and Sample Preservation

Primary sources for dietary DNA metabarcoding include fecal DNA (fDNA, obtained via feces or anal swabs) and gastrointestinal (stomach or intestinal) contents, depending on the target species and research objectives. For accessible species, DNA is most commonly extracted from gut contents, which contain abundant partially digested prey DNA and thus offer high-resolution insights into feeding patterns [32]. However, large-scale dissection sampling can be labor-intensive and time-consuming, and it is unsuitable for rare, protected, or large pelagic species. For such taxa, non-invasive alternatives such as fecal collection or anal swabbing have become practical solutions [33]. These methods minimize harm to the host but can be affected by variables such as fecal freshness and prey digestion status, both of which influence DNA recovery and amplification efficiency [34]. However, they are vulnerable to external DNA contamination, which may compromise data reliability. Therefore, sampling strategies should be tailored to species accessibility and study goals—gut contents for common species, and non-invasive methods for rare or endangered taxa.

Following collection, rapid and appropriate preservation is essential to prevent DNA degradation. Cryopreservation remains the gold standard, with preservation efficiency inversely correlated with temperature. Typical protocols employ freezing at −20 °C to −80 °C, with optimal stabilization achieved through flash-freezing in liquid nitrogen or on dry ice [35,36,37,38,39]. Both liquid nitrogen and ethanol preservation have proven suitable for downstream genomic analyses [40]. When refrigeration is unavailable in the field, storage in 80–100% ethanol is recommended [41,42]. Combining ethanol preservation with cryostorage or buffer solutions can further enhance stability [43,44]. Comparative assessments show that DNeasy-Freeze preservation yields the highest DNA recovery, followed by DNeasy-Ethanol and PW-Freeze methods [45]. Additionally, silica gel desiccation can perform comparably to cryopreservation under certain conditions, such as when applied to specific sample types (e.g., fecal or filter samples), ensuring rapid and complete drying, and when the desiccated samples are stored at stable, low-to-moderate temperatures for short to medium terms, while maintaining community composition and offering a practical alternative in fieldwork [46,47,48,49]. Recent advances also demonstrate the efficacy of lysate buffer-based preservation for stabilizing DNA in variable environments [50].

### 2.2. Primer Selection

Primer selection is a critical determinant of DNA metabarcoding accuracy and should be optimized based on the expected prey spectrum of the focal species [51]. Our literature review of 101 relevant studies summarizes the principal DNA metabarcoding primers employed in dietary analyses (Table 1; Appendix A, Table A1), offering guidance for evidence-based primer choice in future research.

Because fish exhibit diverse feeding habits, particularly omnivorous species consuming prey from multiple taxa, a single primer set often fails to capture the full dietary range. Consequently, many studies employ multiple primer pairs targeting different barcode regions to broaden taxonomic coverage. In contrast, herbivorous fishes with relatively homogeneous diets are often effectively profiled using a single primer set. For example, a study on white dolphins reported that increasing the number of primer pairs significantly improved prey detection rates [6]. Hence, employing multiple primer pairs enhances taxonomic recovery and accuracy, especially for generalist feeders.

Among the primers, mlCOIintF/jgHCO2198 (Figure 1b) was the most prevalent, frequently used, providing broad coverage across metazoans and demonstrating high amplification efficiency [43]. For carnivorous or omnivorous fishes, combining multiple primer sets is strongly recommended to achieve comprehensive dietary profiles. In contrast, filter-feeding species with narrow diets may be sufficiently analyzed with a single primer pair, though using multiple markers can still enhance detection depth. Herbivorous species commonly utilize primers such as CYA359F/CYA781Ra(b) and p23SrV_f1/p23SrV_r1, while the chloroplast trnL marker—widely applied in herbivorous mammals—remains underused but potentially valuable for fish.

A major technical challenge is host DNA contamination, which can obscure prey detection, especially in degraded gut content samples [41,52]. Blocking primers designed to suppress host DNA amplification can mitigate this issue, but may inadvertently inhibit closely related prey taxa [53,54,55]. Thus, empirical optimization and validation are essential to balance host suppression with prey amplification efficiency.

### 2.3. Reference Databases Utilization

Accurate taxonomic identification in dietary DNA metabarcoding relies on robust reference databases [56]. Commonly used repositories include NCBI GenBank, BOLD (Barcode of Life Data System), and Silva [57]. Among these, NCBI offers the broadest taxonomic representation, whereas BOLD focuses on COI sequences from animals, and Silva specializes in ribosomal RNA genes. Beyond public repositories, constructing local reference databases can greatly enhance taxonomic resolution, especially for regionally restricted or poorly represented taxa [56]. These databases, built from locally collected prey tissues and integrated with curated public records, help capture regional biodiversity and reduce misidentifications caused by incomplete or biased global datasets.

When a single database is insufficient, integrating multiple databases can enhance reliability. Among the 101 reviewed studies, 27.6% employed multiple resources. Within this subset, the most common strategy involved combining a general public database with BOLD, particularly GenBank + BOLD. Other combinations, such as rRNA-based databases (e.g., Silva) used in conjunction with BOLD, were reported less frequently, while a limited number of studies adopted hybrid systems that integrated public repositories with locally curated reference libraries. Despite the additional effort required to develop locally curated reference databases, studies incorporating both BOLD and local references have been shown to achieve notably improved prey identification accuracy [58]. Therefore, best practice entails initial annotation with local databases, followed by cross-validation using public repositories to improve completeness and precision.

### 2.4. Bioinformatics Analysis Process

The robustness of bioinformatics analysis is pivotal for accurate interpretation of DNA metabarcoding data [56]. Commonly used tools in fish dietary studies include QIIME, USEARCH, Mothur, Sickle, OBITools, FASTP, and DADA2 [39,42,59,60,61]. These analytical frameworks primarily utilize two distinct bioinformatics strategies: Operational Taxonomic Unit (OTU) classification and Amplicon Sequence Variant (ASV) analysis, which differ primarily in clustering stringency and error-correction principles. Processing begins with trimming primers, host sequences, and barcodes, followed by stringent quality filtering to obtain high-confidence reads. Sequences are then clustered into OTUs (97–99% similarity) or denoised into ASVs (100% resolution) [62]. Among 101 studies reviewed, 63.7% used OTU-based, 25.5% ASV-based, and 2.9% hybrid strategies (Figure 1c). ASV approaches generally outperform OTUs due to finer taxonomic resolution, advanced error correction, and enhanced reproducibility, while eliminating the need for arbitrary similarity thresholds that often obscure true biological variation. However, they may exclude low-abundance sequences during noise reduction and over-split taxa owing to intra-genomic variation. Despite ongoing debate, ASVs are increasingly favored for their higher sensitivity and standardization potential.

Taxonomic assignment is performed by comparing representative OTU/ASV sequences to reference databases using algorithms such as BLAST, RDP, or QIIME2-classify-sklearn, as well as tools like UCLUST, ecoTag, and INSECT [6,39,61]. Accuracy depends on marker choice, database completeness, and classification method [56]. Empirical evidence indicates that employing multiple annotation algorithms with cross-validation markedly improves the reliability of taxonomic assignments [63]. Finally, annotated results should be manually curated for biological plausibility, ensuring consistency with known species distributions and ecological characteristics.

## 3. Application of DNA Metabarcoding in Fish Dietary Studies

**Figure 3:**
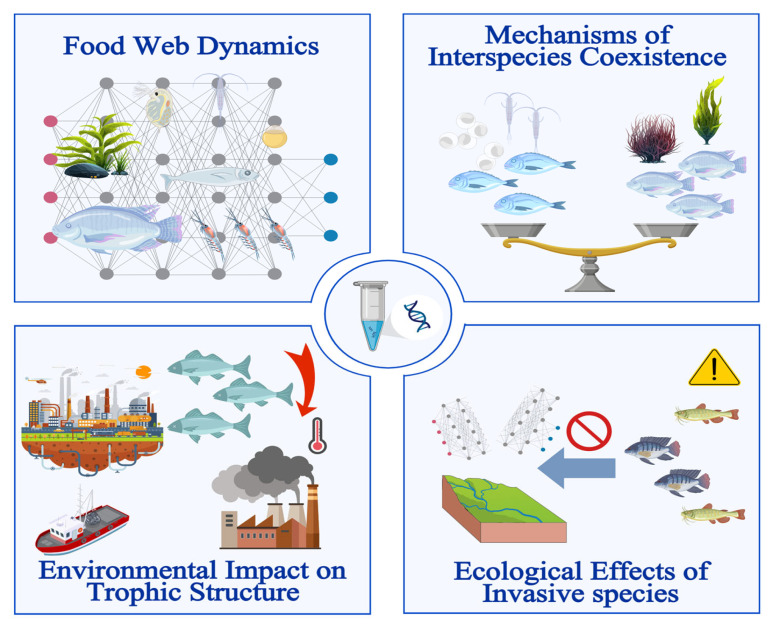
Applications of DNA metabarcoding in fish trophic ecology. Conceptual framework illustrating the major applications of DNA metabarcoding in fish trophic studies, including elucidation of food web relationships, quantification of environmental drivers, investigation of species interactions and coexistence, and assessment of invasive species impacts.

Fish diet analysis is far more than a simple inventory of prey; it is a gateway to understanding species-environment interactions, food web architecture, and the stability of aquatic ecosystems [34]. While traditional visual stomach content analysis often suffers from “taxonomic blurring” due to prey digestion, DNA metabarcoding provides the taxonomic resolution necessary to resolve complex ecological questions (Figure 3; Table 2). The “added benefit” of this molecular approach is clearly demonstrated in studies that integrated both methods. For instance, in an analysis of Silver croaker (*Pennahia argentata*) diet, Kim et al. (2022) [64] identified 44 prey species via DNA metabarcoding, compared to only 7 through morphological examination. Similarly, Li et al. (2024) [65] found that while traditional methods identified 8 species in Masu salmon (*Oncorhynchus masou*), DNA metabarcoding significantly expanded this to 45 species. These cases illustrate how molecular tools resolve highly digested remains, providing a far more comprehensive picture of fish trophic interactions.

### 3.1. Elucidating Food Web Relationships

The high sensitivity of DNA metabarcoding has revolutionized food web topology by uncovering previously “invisible” trophic links. In aquatic systems, many critical interactions involving soft-bodied organisms (e.g., jellyfish, larval stages, or cryptic invertebrates) are systematically underestimated by visual methods. Molecular tools effectively rectify this bias, revealing a more interconnected and robust ecosystem than previously realized.

Multi-primer DNA metabarcoding has proven to be highly effective in resolving trophic interactions and energy flow within food webs [66]. For example, Lu et al. (2023) [67] combined DNA metabarcoding with network analysis to investigate trophic relationships among carnivorous mammals across three regions of the Tibetan Plateau, identifying sage grouse (*Ochotona* spp.), rock goat (*Pseudois nayaur*), and yak (*Bos grunniens*) as key prey species. This study illustrates the value of integrating molecular dietary analysis with network approaches to reconstruct food webs, identify functionally important species, and elucidate mechanisms of species coexistence. Similar approaches applied to fish communities allow detailed assessment of interspecific energy transfer, niche partitioning, and the roles of keystone species, providing critical insights for maintaining aquatic biodiversity and ecosystem stability [67,68].

By resolving cryptic or highly digested “weak links” in a food web, DNA metabarcoding can reveal a much higher degree of connectivity than previously estimated. These numerous weak interactions often act as biological buffers, preventing the destabilization of the entire system when a single primary prey species declines. Therefore, molecular-based food web reconstructions offer a more realistic baseline for assessing the resilience of aquatic ecosystems to external shocks.

### 3.2. Quantifying Trophic Plasticity and Environmental Drivers

**Figure 4:**
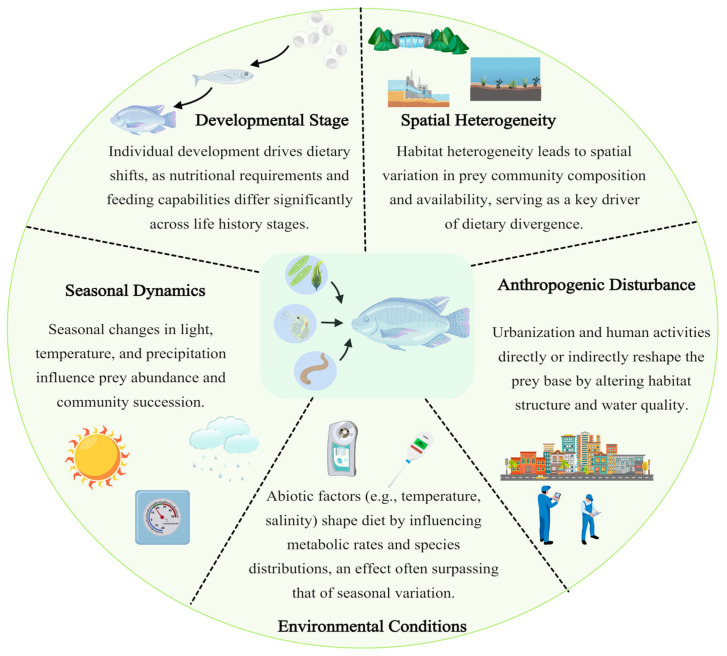
Conceptual framework of fish trophic plasticity and its environmental drivers. Five key factors influencing trophic plasticity in fish are illustrated: individual developmental stage, spatial heterogeneity in habitat, anthropogenic disturbance, abiotic environmental factors, and seasonal variations.

DNA metabarcoding enables fine-scale assessment of fish feeding selectivity and its spatiotemporal variability, enhancing understanding of trophic plasticity under varying ecological and environmental contexts (Figure 4; Table 2). For instance, 18S rDNA metabarcoding revealed ontogenetic dietary shifts in Japanese halfbeak (*Hyporhamphus sajori*), with juveniles consuming a diverse diet dominated by Arthropoda (45.3%) and algae (Chlorophyta 20.3%, Bacillariophyta 12.3%, Pyrrophyta 12.4%), whereas larger individuals relied almost exclusively on Arthropoda (97.2%) [69]. Such findings highlight the importance of tailoring feeding strategies to developmental stages. Similarly, spatial differences in diets of European pilchard (*Sardina pilchardus*) and abalone provide insights into prey availability and aquaculture management [70]. Urbanization and human disturbance further affect fish diets; studies indicate that environmental conditions often outweigh seasonal variation in shaping prey selection, with potential consequences for community structure and ecosystem health [71,72].

Trophic plasticity, as evidenced by these ontogenetic and spatial shifts, is not merely a behavioral trait but a vital survival mechanism in fluctuating aquatic environments. High-resolution DNA data allow us to pinpoint “nutritional bottlenecks”—critical life stages where a fish’s survival depends on a narrow range of specific prey. Understanding these fine-scale dietary requirements is essential for predicting how species will respond to habitat degradation.

### 3.3. Mechanisms of Species Interactions and Coexistence

Ecosystem stability depends on complex networks of interactions including competition, predation, mutualism, symbiosis, and parasitism [72]. Elucidating the mechanisms underlying these interactions is central to understanding how species coexist within diverse fish communities. DNA metabarcoding facilitates simultaneous detection of multiple taxa across trophic levels, enabling the study of interspecific interactions and coexistence strategies [66]. In aquatic systems, food and habitat resources are often limited, and coexistence among sympatric fish species is frequently mediated by a combination of resource partitioning, behavioral differentiation, and spatial or temporal segregation. For example, analyses of benthic coral reef fish revealed significant differences in prey selection among four sympatric species, indicating resource partitioning and spatial segregation as mechanisms promoting coexistence [42].

Coexistence is not just about sharing resources, but about a high degree of micro-resource segregation. The core advantage of DNA metabarcoding lies in its ability to resolve “taxonomic blurring,” a common limitation in visual analysis that often underestimates niche partitioning. Ultimately, the stability and maintenance of high-diversity fish communities rely on an intricate web of specialized links, where even subtle dietary differences represent a sophisticated evolutionary strategy to minimize niche overlap and maintain long-term coexistence.

### 3.4. Invasive Species and Ecosystem Impacts

Invasive species pose substantial challenges to aquatic ecosystems. DNA metabarcoding has been applied to assess their dietary ecology and potential ecological impacts. In Puerto Rico, analysis of gut contents of invasive lionfish (*Pterois volitans*) revealed predation on native species such as queen parrotfish (*Scarus vetula*), stoplight parrotfish (*Sparisoma viride*), and striped parrotfish (*Scarus iseri*), highlighting potential threats to native populations [73]. Similarly, comparative analyses in Lake Michigan showed that invasive fishes, such as alewife (*Alosa pseudoharengus*) and rainbow smelt (*Osmerus mordax*), have successfully integrated into existing food webs [58]. These species occupy multiple trophic levels and compete directly with native species, including bloater (*Coregonus hoyi*), ninespine stickleback (*Pungitius pungitius*), and slimy sculpin (*Cottus cognatus*). These findings underscore the utility of DNA metabarcoding in monitoring invasive species and evaluating their effects on ecosystem structure and function.

Beyond simply documenting what invasive species eat, DNA metabarcoding serves as an “early warning system” for trophic disruption. By detecting the consumption of native keystone species or rare taxa at very early stages of invasion, molecular tools provide managers with a critical window for intervention. Furthermore, comparing the diet of invaders with native competitors allows us to quantify “trophic niche displacement,” where native species are squeezed into narrower or less productive niches. This functional insight is far more valuable for ecosystem management than simple presence-absence data, as it links individual predation events to broad-scale community restructuring.

**Table 2:** Representative case studies exploring key areas in animal dietary ecology.

Study Species	Ecosystem	Target Analysis	Major Findings
Carnivorous mammals	Alpine ecosystem	Predator-prey networks, identification of key prey	Identified pika, blue sheep, and yak as key prey; reconstructed spatially explicit food webs and coexistence mechanisms [67].
Fish communities	Various aquatic ecosystems	Energy flow, niche partitioning, keystone species	Quantified energy transfer pathways and the role of weak trophic links in maintaining ecosystem stability [68].
Japanese halfbeak	Coastal waters	Ontogenetic dietary shifts	Diet shifted from diverse (arthropods, algae) in juveniles to almost exclusive arthropod consumption in adults, demonstrating high ontogenetic plasticity [69].
Abalone	Coastal reefs	Spatial diet variability	Revealed significant geographical differences in diet composition linked to local prey availability using stable isotope analysis [70].
Four sympatric benthic reef fish	Coral reef ecosystem	Resource partitioning and niche segregation	Found significant interspecific differences in prey composition, indicating fine-scale resource partitioning facilitating coexistence [42].
*Pennahia argentata*	Coastal waters	Interspecific interactions, food web structure	Elucidated complex competitive and facilitative interactions within diverse communities, clarifying mechanisms of coexistence [64].
Invasive lionfish	Coral reefs, Puerto Rico	Predation impact on native fish fauna	Confirmed predation on ecologically important native parrotfishes, highlighting a threat to reef ecosystem function [73].
Invasive species and native fish	Laurentian Great Lakes	Food web integration and trophic competition	Documented broad integration into native food webs and direct resource competition with native fishes across multiple trophic levels [58].

## 4. Current Challenges and Future Solutions

### 4.1. From Bias to Precision: Improving Data Reliability

**Figure 5:**
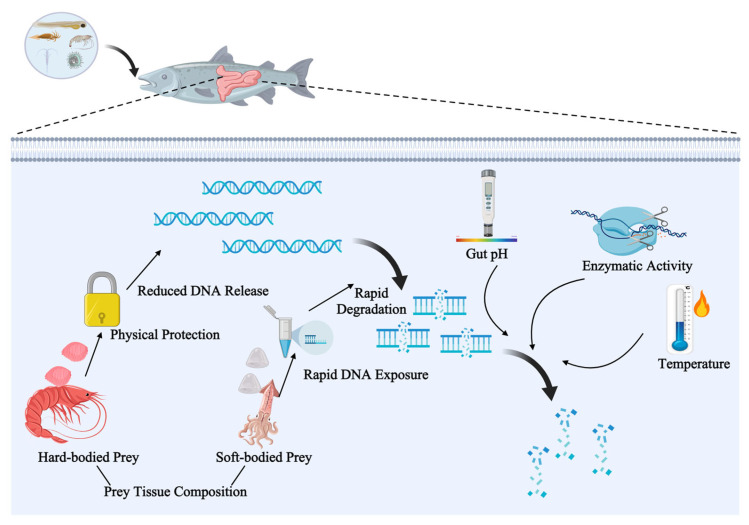
Factors influencing the degradation of dietary DNA in the fish gut. DNA persistence is co-regulated by factors including enzymatic activity, pH, temperature, and prey tissue composition. Hard-bodied prey provide physical protection against degradation, whereas soft-bodied prey undergo rapid exposure. This differential degradation creates an integrated signal over variable time windows, representing a critical bias in DNA metabarcoding-based diet interpretation.

Current DNA metabarcoding in fish research is hindered by both biological and technical factors. Biological constraints, such as differential DNA degradation (Figure 5) in the gut and the dominance of predator DNA, often lead to biased diet profiles [52]. Hard-bodied prey (e.g., crustaceans, mollusks) often yield different detection probabilities than soft-bodied prey, producing a potentially biased view of diet composition. Moreover, DNA persistence varies among species and individuals due to differences in metabolism and digestion rates [74]. As a result, gut DNA represents an integrated signal over a variable digestion window rather than a strict record of immediate feeding events, a limitation that also affects traditional visual stomach content analysis. While this biological heterogeneity can fundamentally limit the temporal precision and taxonomic completeness of diet reconstruction [74,75], such biases can be partially mitigated by sampling a larger number of individuals to capture a broader population-level signal.

Simultaneously, every analytical step—from primer design to bioinformatics—can introduce systematic biases, representing a broader challenge inherent to all DNA metabarcoding applications. Primer-template mismatches cause preferential amplification of certain taxa, while highly degraded DNA fragments from soft tissues may be overrepresented. Although shorter amplicons enhance detectability, they reduce taxonomic resolution [54], and incomplete or misannotated reference databases may propagate identification errors [57]. PCR stochasticity and barcode mismatches can further produce spurious reads or inflate rare taxa [52,76].

To address these, future efforts must move beyond generic protocols toward fish-specific innovations. Developing regional, habitat-specific reference libraries is essential to reduce misidentification. Furthermore, employing host-blocking primers or CRISPR-based depletion of predator DNA can effectively mitigate the “predator-masking” effect, ensuring the detection of trace prey sequences even in samples with a high host background. Emerging technologies like Nanopore sequencing also allow for rapid, on-site assessment, reducing the risk of DNA degradation during long-term sample storage in remote field studies.

### 4.2. Beyond Species Lists: Quantitative and Multi-Method Integration

The primary objective of DNA metabarcoding-based trophic studies is to facilitate sophisticated ecological interpretation, moving beyond simple taxonomic inventories to characterize food web dynamics. However, a major challenge to this interpretation is that sequence counts cannot be directly translated into trophic strength or energy flow without accounting for prey DNA copy number, digestibility, and turnover rates. Moreover, fish diets are highly dynamic, varying with ontogeny, reproductive status, and habitat conditions. While dietary DNA metabarcoding captures a snapshot of recent feeding, it provides limited information on prey biological traits, such as size, developmental stage, or nutritional value. Indirect or secondary predation further complicate interpretation. DNA traces detected in a predator’s gut may originate not from its direct prey, but from prey-of-prey (e.g., when a fish consumes a crustacean that recently ingested phytoplankton). These secondary signals blur trophic boundaries and can inflate apparent dietary breadth. Similarly, environmental or non-dietary DNA contamination—from ambient water, sediment particles, or prey mucus adhering to gill or gut surfaces—may lead to false positives.

The solution lies in a multi-proxy integrative framework. By combining molecular data with traditional morphological analysis, researchers can recover prey size and biomass information. Meanwhile, integrating stable isotope analysis (SIA) or fatty acid profiling provides a long-term view of energy flow that complements the short-term “snapshot” of DNA data. Specifically, DNA metabarcoding reflects recent feeding events occurring over hours to days, whereas stable isotope signatures integrate assimilated diet over longer timescales ranging from weeks to months. When interpreted together, short-term DNA signals help identify taxonomically resolved prey sources, while long-term isotope data constrain trophic position and energy pathways, allowing transient feeding events to be distinguished from sustained dietary contributions. This synergy transforms DNA metabarcoding from a descriptive method into a robust quantitative tool capable of modeling the complexity of aquatic food webs and their response to environmental change.

### 4.3. Strategic Applications in Fisheries and Conservation

Translating these high-resolution dietary datasets into actionable management strategies represents the next frontier for the field. Overcoming methodological hurdles is only the first step; the broader utility of DNA metabarcoding lies in its ability to support evidence-based fisheries management. The absence of standardized protocols across studies could hamper comparability and synthesis. Variations in sampling design, DNA extraction methods, primer sets, and bioinformatic pipelines (e.g., OTU- vs. ASV-based analyses) can produce markedly different community profiles. These inconsistencies hinder the construction of large-scale trophic networks and meta-analyses. Future research should prioritize methodological harmonization—adopting FAIR-compliant metadata standards (e.g., MIxS) and integrating DNA metabarcoding results with complementary ecological datasets (e.g., stable isotopes, fatty acids, trait-based models)—to achieve reproducible and ecologically interpretable dietary reconstructions.

Large-scale, high-resolution, and integrated trophic datasets are critical for identifying key prey species for endangered fish, assessing the impact of invasive species, and predicting food web responses to climate change. By coupling DNA-based dietary reconstruction with predictive ecological modeling, DNA metabarcoding is set to evolve into a forward-looking paradigm for maintaining ecosystem resilience and informing sustainable exploitation policies. This transition from a laboratory diagnostic to a management tool will be pivotal for the future of aquatic conservation.

## 5. Conclusions

DNA metabarcoding has opened new avenues for exploring fish trophic ecology, offering a powerful lens into the complexity of aquatic food webs. Yet its full ecological potential depends on bridging methodological, interpretive, and integrative gaps. Moving forward, combining technological innovation with ecological theory will be essential to transform sequence data into meaningful insights for biodiversity assessment and ecosystem management.

## Figures and Tables

**Table 1:** List of primers for DNA metabarcoding that were used in more than two studies in this review.

Target Taxa	Primer Names	Sequences	Target Region	Fragment Length (bp)	Frequency
Metazoan	mlCOIintF	GGNGGNTTYGGNAAYTG	COI	~313	44
	jgHCO2198	GGRTGNCCRAARAAYCA			
Eukaryote	TAReuk454FWD1	CCAGCASCYGCGGTAATTCC	18S V4	~380	5
	TAReukREV3	ACTTTCGTTCTTGATYRA			
Eukaryote	528F	GCGGTAATTCCAGCTCCAA	18S V4	~350	5
	706R	AATCCRAGAATTTCACCTCT			
Arthropod	ZBJ-ArtF1c	AGATATTGGAACWTTATATTTTATTTTTGG	COI	~200	5
	ZBJ-ArtR2c	WACTAATCAATTWCCAAATCCTAA			
Fish	MiFish-U-F	GTCGGTAAAACTCGTGCCAGC	12S	~171	4
	MiFish-U-R	CATAGTGGGGTATCTAATCCCAGTTTG			
Phytoplankton	1380F	CCCTGCCHTTTGTACACAC	18S V9	~200	3
	1510R	CCTTCYGCAGGTTCACCTAC			
Algae	p23SrV_f1	GGACAGAAAGACCCTATGAA	23S	~430	3
	p23SrV_r1	TCAGCCTGTTATCCCTAGAG			

## Data Availability

No new data were created or analyzed in this study. Data sharing is not applicable to this article.

## Appendix A

**Table A1:** Complementary list of primers: sequences reported in two or fewer studies.

Target Taxa	Primer Names	Sequences	Target Region	Fragment Length (bp)	Frequency
Fish	teleo_F	ACACCGCCCGTCACTCT	12S	<100	2
	teleo_R	CTTCCGGTACACTTACCATG			
Chordata	Chord_16S_F	CGAGAAGACCCTRTGGAGCT	16S	~120	1
	Chord_16S_R	CCTNGGTCGCCCCAAC			
Cephalopoda	Ceph_16S_F	GACGAGAAGACCCTAWTGAGCT	16S	~260–310	1
	Ceph_16S_R	AAATTACGCTGTTATCCCT			
Cephalopoda	Sceph_F	GCTRGAATGAATGGTTTGAC	16S	90–110	1
	Sceph_R	TCAWTAGGGTCTTCTCGTCC			
Crustacea	Crust_F	GGGACGATAAGACCCTATA	16S	140–190	2
	Crust_R	ATTACGCTGTTATCCCTAAAG			
Teleost	Fish16S_F	GACCCTATGGAGCTTTAGAC	16S	200	2
	Fish16S_R	CGCTGTTATCCCTADRGTAACT			
Eukaryote	319F	ACTCCTACGGGAGGCAGCAG	16S	250	1
	806R	GGACTACHVGGGTWTCTAAT			
Bacterium/Cyanobacteria	Bact-0341	CCTACGGGNGGCWGCAG	16S V3–V4	/	1
	Bact-0758	GACTACHVGGGTATCTAATCC			
Universal primers	18SUni_F	GCCAGTAGTCATATGCTTGTCT	18S	350–420	2
	18SUni_R	GCCTGCTGCCTTCCTT			
Eukaryote	Euk_18S_F	GTACACACCGCCCGTC	18S	~260	1
	Euk_18S_R	TGATCCTTCTGCAGGTTCACCTAC			
Cephalopod	Ceph18S_F	CGCGGCGCTACATATTAGAC	18S	200	1
	Ceph18S_R	GCACTTAACCGACCGTCGAC			
Eukaryote	574YF	TCGTCGGCAGCGTCAGATGTGTATAAGAGACAGCGGTAAYTCCAGCTCYV	18S	167–569	2
	1132R	GTCTCGTGGGCTCGGAGATGTGTATAAGAGACAGCCGTCAATTHCTTYAART			
Eukaryote	18S_allshorts_F	TTTGTCTGSTTAATTSCG	18S	~110	1
	18S_allshorts_R	GCAATAACAGGTCTGTG			
Metazoan	SSUF04_F	GCTTGTCTCAAAGATTAAGCC	18S V1–V2	356	1
	SSURmod_R	CCTGCTGCCTTCCTTRGA			
Metazoan	SSU_F04	GCTTGTCTCAAAGATTAAGCC	18S V1–V2	~450	1
	SSU_R22	CCTGCTGCCTTCCTTRGA			
Marine invertebrates	18S_uni_1F	GCCAGTAGTCATATGCTTGTCT	18S V1–V3	~340–420	2
	18S_uni_400R	GCCTGCTGCCTTCCTT			
Eukaryote	E572F	CYGCGGTAATTCCAGCTC	18S V4	~400	1
	E1009R	AYGGTATCTRATCRTCTTYG			
Eukaryote	S616F	TTAAAAAGCKKCGTAGTTG	18S V4	/	1
	V4R	ACTTTCGTTCTTGAT			
Eukaryote	Uni18SF	AGGGCAAKYCTGGTGCCAGC	18S V4	400–600	1
	Uni18SR	GRCGGTATCTRATCGYCTT			
Eukaryote	D512F	ATTCCAGCTCCAATAGCG	18S V4	390–410	1
	D978R	GACTACGATGGTATCTAATC			
Eukaryote	547F	CCAGCASCYGCGGTAATTCC	18S V4	~420	2
	V4R	ACTTTCGTTCTTGATYRA			
Eukaryote	Uni18SF2	CTTAATTTGACTCAACACGG; CTTAATTTGACTCAACACGG	18S V5–V7	400–600	1
	Uni18SR2	TAGCGACGGGCGGTGTGTAC; TAGCGACGGCGGTGTGTAC			
Eukaryote	1389F	TTGTACACACCGCCC	18S V9	~130	2
	1510R	CCTTCYGCAGGTTCACCTAC			
Eukaryote	Euk1319F	GTACACACCGCCCGTC	18S V9	~200	2
	EukBr	TGATCCTTCTGCAGGTTCACCTAC			
Fish	FishF_lh1	TAATRATYTTYTTYATRGTNATRCC	COI	~130	1
	FishR_lh1	CTYATRTTRTTYATICGIGG			
Fish	FishF_lh2	ACHGGNTGRACNGTNTAYCCNCC	COI	~300	1
	FishR2_t1/FR1d_t1	ACTTCAGGGTGACCGAAGAATCAGAA/ACCTCAGGGTGTCCGAARAAYCARAA			
Fish	AquaF2_t1	ATCACRACCATCATYAAYATRAARCC	COI	184	1
	C_FishR1_t1	CAGGAAACAGCTATGACACCTCAGG GTGTCCGAARAAYCARAA			
Fish	FISH_BCL	TCAACYAATCAYAAAGATATYGGCAC	COI	/	1
	FISH_BCH	ACTTCYGGGTGRCCRAARAATCA			
Arthropod	Ill_B_F	CCIGAYATRGCITTYCCICG	COI	~300	1
	ArR5	GTRATIGCICCIGCIARIACIGG			
Metazoan	COIMISQF1	ATNGGNGGNTTYGGNAA	COI	463	2
	COIMISQR1	TANACYTCNGGRTGNCC			
Invertebrate	LCO1490	GGTCAACAAATCATAAAGATATTGG	COI	710	1
	HCO2198	TAAACTTCAGGGTGACCAAAAAATCA			
Metazoan	LoboR1	TAAACYTCWGGRTGWCCRAARAAYCA	mtCOI	~658	2
	LCO1490	GGTCAACAAATCATAAAGATATTGG			
Cyanobacteria	CYA359F	GGGGAATYTTCCGCAATGGG	16S	~450	1
	CYA781Ra/CYA781Rb	GACTACTGGGGTATCTAATCCCATT/GACTACAGGGGTATCTAATCCCTTT			
Diatom	Diat_rbcL708F_2	AGGTGAAGTTAAAGGTTCWTAYTTAAA	rbcL	312	1
	R3_1	CCTTCTAATTTACCWACWACTG			
Plant	RbcL-Z1aF	CGTCCTTTGTAACGATCAAG	rbcL	~230	1
	RbcL-hp2R	GGACTTATGACATGAAAAATCATCGTTG			
Actinozoa/Demospongiae	scl58SF	GARTCTTTGAACGCAAATGGC	ITS2	400	1
	scl28SR	GCTTATTAATATGCTTAAATTCAGCG			
Mollusca/Gastropoda	C_GasF1_t1	TGTAAAACGACGGCCAGTTTTCAACAAACCATAARGATATTGG	COI	~283	1
	MGasR1_t1	CAGGAAACAGCTATGACACTTCWGGRTGHCCRAARAATCARAA			
Amphipoda	Prey_AnnelidF1_t1	/	COI	~193	2
	Prey_AmphipodR1_t1	/			

(“/” Indicates no information found).

## References

[1] Nunes L., Morais R., Longo G., Sabino J., Floeter S. (2020). Habitat and community structure modulate fish interactions in a neotropical clearwater river. Neotrop. Ichthyol..

[2] Zhang Y., Yu Z., Xu Q., Li X., Zhu S., Li J. (2022). Regionally divergent patterns of grass carp relative abundance. feeding habits and trophic niches in the subtropical Pearl River basin. Aquat. Ecol..

[3] Ford B.M., Roberts J.D. (2019). Evolutionary histories impart structure into marine fish heterospecific co-occurrence networks. Glob. Ecol. Biogeogr..

[4] Fernández-Cisternas I., Majlis J., Ávila-Thieme M.I., Lamb R.W., Pérez-Matus A. (2021). Endemic species dominate reef fish interaction networks on two isolated oceanic islands. Coral Reefs.

[5] Freestone A.L., Torchin M.E., Jurgens L.J., Bonfim M., López D.P., Repetto M.F., Schlöder C., Sewall B.J., Ruiz G.M. (2021). Stronger predation intensity and impact on prey communities in the tropics. Ecology.

[6] Zhang J., Yang H., Ma M., Pu T., Yin X. (2023). Predator-mediated diversity of stream fish assemblages in a boreal river basin. China. Sci. Rep..

[7] Villéger S., Brosse S., Mouchet M., Mouillot D., Vanni M.J. (2017). Functional ecology of fish: Current approaches and future challenges. Aquat. Sci..

[8] Benkwitt C.E., Wilson S.K., Graham N.A.J. (2020). Biodiversity increases ecosystem functions despite multiple stressors on coral reefs. Nat. Ecol. Evol..

[9] Du X.-X., Tian S.-Q., Wang J.-Q., Wang Z.-H., Gao C.-X. (2018). Spatial and temporal variations in fish community offshore southern Zhejiang Province, East China Sea. J. Dalian Ocean. Univ..

[10] Yang H. (2021). Role of fish as bioindicators of aquatic ecosystem health. J. Aquat. Ecol..

[11] Embayments O. (2008). The Role of Environmental Characteristics on Fish Community Structure and Food Web Interactions in Lake. https://www.proquest.com/docview/304631376/abstract/D612035D878D4E72PQ/1.

[12] Hyslop E.J. (1980). Stomach contents analysis—A review of methods and their application. J. Fish Biol..

[13] Motta P.J., Clifton K.B., Hernandez P., Eggold B.T. (1995). Ecomorphological correlates in ten species of subtropical seagrass fishes: Diet and microhabitat utilization. Environ. Biol. Fishes.

[14] Piet G.J. (1998). Ecomorphology of a size-structured tropical freshwater fish community. Environ. Biol. Fishes.

[15] Pethybridge H., Daley R.K., Nichols P.D. (2011). Diet of demersal sharks and chimaeras inferred by fatty acid profiles and stomach content analysis. J. Exp. Mar. Biol. Ecol..

[16] Xia Y., Li Y., Zhu S., Li J., Li S., Li X. (2020). Individual dietary specialization reduces intraspecific competition, rather than feeding activity, in black amur bream (*Megalobrama terminalis*). Sci. Rep..

[17] Antil S., Abraham J.S., Sripoorna S., Maurya S., Dagar J., Makhija S., Bhagat P., Gupta R., Sood U., Lal R. (2023). DNA barcoding, an effective tool for species identification: A review. Mol. Biol. Rep..

[18] Corse E., Costedoat C., Chappaz R., Pech N., Martin J.-F., Gilles A. (2010). A PCR-based method for diet analysis in freshwater organisms using 18S rDNA barcoding on faeces. Mol. Ecol. Resour..

[19] Wang S., Jin B., Qin H., Sheng Q., Wu J. (2015). Trophic dynamics of filter feeding bivalves in the Yangtze estuarine intertidal marsh: Stable isotope and fatty acid analyses. PLoS ONE.

[20] Xu Y., Jing Y., Zhou J., Long R., Meng J., Yang Y., Luo Y. (2023). Age, growth, and energy storage of the subterranean fish Triplophysa rosa (Cy-priniformes: Nemacheilidae) from Chongqing, China. BMC Ecol. Evol..

[21] Budge Suzanne M., Penney Sarah N., Lall Santosh P. (2012). Estimating diets of Atlantic salmon (*Salmo salar*) using fatty acid signature analyses; validation with controlled feeding studies. Can. J. Fish. Aquat. Sci..

[22] Barbato M., Kovacs T., Coleman M.A., Broadhurst M.K., de Bruyn M. (2019). Metabarcoding for stomach-content analyses of Pygmy devil ray (*Mobula kuhlii* cf. eregoodootenkee): Comparing tissue and ethanol preservative-derived DNA. Ecol. Evol..

[23] Adamowicz S.J., Boatwright J.S., Chain F., Fisher B.L., Hogg I.D., Leese F., Lijtmaer D.A., Mwale M., Naaum A.M., Pochon X. (2019). Trends in DNA barcoding and metabarcoding. Genome.

[24] Deiner K., Walser J., Mchler E., Altermatt F. (2015). Choice of capture and extraction methods affect detection of freshwater biodiversity from environmental DNA. Biol. Conserv..

[25] Zizka V.M.A., Leese F., Peinert B., Geiger M.F. (2019). DNA metabarcoding from sample fixative as a quick and voucher-preserving biodiversity assessment method. Genome.

[26] Huang P.-Y., Poon E., Chan L.Y., Chan D.T.C., Huynh S., So I., Sung Y.-H., Sin S. (2022). Dietary diversity of multiple shorebird species in an Asian subtropical wetland unveiled by DNA metabarcoding. Environ. DNA.

[27] Stenhouse E.H., Bellamy P., Kirby W., Vaughan I.P., Drake L.E., Marchbank A., Workman T., Symondson W.O.C., Orozco-terWengel P. (2023). Multi-marker DNA metabarcoding reveals spatial and sexual variation in the diet of a scarce woodland bird. Ecol. Evol..

[28] Symondson W.O.C. (2002). Molecular identification of prey in predator diets. Mol. Ecol..

[29] Valentini A., Pompanon F., Taberlet P. (2009). DNA barcoding for ecologists. Trends Ecol. Evol..

[30] Kircher M., Kelso J. (2010). High-throughput DNA sequencing—Concepts and limitations. Bioessays.

[31] Taberlet P., Coissac E., Pompanon F., Brochmann C., Willerslev E. (2012). Towards next-generation biodiversity assessment using DNA metabarcoding. Mol. Ecol..

[32] Cai W.-J., Zhang Y., Li X., Liu H., Chen L., Yang T., Wang J., Zhou Z. (2023). Intestinal microstructure and dietary analysis based on macrobarcode of *Gymnocypris przewalskii*. Acta Hydrobiol. Sin..

[33] Van Zinnicq Bergmann M.P.M., Postaire B.D., Gastrich K., Heithaus M.R., Hoopes L.A., Lyons K., Papastamatiou Y.P., Schneider E.V.C., Strickland B.A., Talwar B.S. (2021). Elucidating shark diets with DNA metabarcoding from cloacal swabs. Mol. Ecol. Resour..

[34] Liu M., Clarke L.J., Baker S.C., Jordan G.J., Burridge C.P. (2020). A practical guide to DNA metabarcoding for entomological ecologists. Ecol. Entomol..

[35] Yoon T.-H., Kang H.-E., Lee S.-R., Lee J.-B., Baeck G.W., Park H., Kim H.-W. (2017). Metabarcoding analysis of the stomach contents of the Antarctic Toothfish (*Dissostichus mawsoni*) collected in the Antarctic Ocean. Peerj.

[36] Sun P., Ling J., Zhang H., Tang B., Jiang Y. (2021). Diet composition and feeding habits of black sea bream (*Acanthopagrus schlegelii*) in Xiangshan Bay based on high-throughput sequencing. Acta Ecol. Sin..

[37] Xia Y., Liu Q., Zhu S., Li Y., Li X., Li J. (2022). Do changes in prey community in the environment affect the feeding selectivity of silver carp (*Hypophthalmichthys molitrix*) in the Pearl River, China?. Sustainability.

[38] Coker D.J., DiBattista J.D., Stat M., Arrigoni R., Reimer J., Terraneo T.I., Villalobos R., Nowicki J.P., Bunce M., Berumen M.L. (2023). DNA metabarcoding confirms primary targets and breadth of diet for coral reef butterflyfishes. Coral Reefs.

[39] Wu P., Wang T., Liu Y., Li C., Xiao Y., Xu S., Han T., Lin L., Quan Q. (2022). Differences in macroalgal consumption by eight herbivorous coral reef fishes from the Xisha Islands, China. Front. Mar. Sci..

[40] Han H., Zhou X., Gao S., Pang B. (2016). A new preservation method of grasshopper samples for genomic DNA extraction. China Plant Prot..

[41] Su M., Liu H., Liang X., Gui L., Zhang J. (2017). Dietary analysis of marine fish species: Enhancing the detection of prey-specific DNA sequences via high-throughput sequencing using blocking primers. Estuaries Coasts.

[42] Brandl S.J., Casey J.M., Meyer C.P. (2020). Dietary and habitat niche partitioning in congeneric cryptobenthic reef fish species. Coral Reefs.

[43] Leray M., Yang J.Y., Meyer C.P., Mills S.C., Agudelo N., Ranwez V., Boehm J.T., Machida R.J. (2013). A new versatile primer set targeting a short fragment of the mitochondrial COI region for metabarcoding metazoan diversity: Application for characterizing coral reef fish gut contents. Front. Zool.

[44] Waraniak J.M., Marsh T.L., Scribner K.T. (2019). 18S rRNA metabarcoding diet analysis of a predatory fish community across seasonal changes in prey availability. Ecol. Evol..

[45] Hinlo R., Gleeson D., Lintermans M., Furlan E. (2017). Methods to maximise recovery of environmental DNA from water samples. PLoS ONE.

[46] Majaneva M., Diserud O.H., Eagle S.H.C., Boström E., Hajibabaei M., Ekrem T. (2018). Environmental DNA filtration techniques affect recovered biodiversity. Sci. Rep..

[47] Wilcox T.M., McKelvey K.S., Young M.K., Engkjer C., Lance R.F., Lahr A., Eby L.A., Schwartz M.K. (2020). Parallel, targeted analysis of environmental samples via high-throughput quantitative PCR. Environ. DNA.

[48] Allison M.J., Round J.M., Bergman L.C., Mirabzadeh A., Allen H., Weir A., Helbing C.C. (2021). The effect of silica desiccation under different storage conditions on filter-immobilized environmental DNA. BMC Res. Notes.

[49] Plante F., Bourgault P., Dubois Y., Bernatchez L. (2021). Environmental DNA as a detection and quantitative tool for stream-dwelling salamanders: A comparison with the traditional active search method. Environ. DNA.

[50] Takahashi M., Saccò M., Kestel J.H., Nester G., Campbell M.A., van der Heyde M., Heydenrych M.J., Juszkiewicz D.J., Nevill P., Dawkins K.L. (2023). Aquatic environmental DNA: A review of the macro-organismal biomonitoring revolution. Sci. Total Environ..

[51] Alberdi A., Aizpurua O., Gilbert M.T.P., Bohmann K. (2018). Scrutinizing key steps for reliable metabarcoding of environmental samples. Methods Ecol. Evol..

[52] Deagle B.E., Thomas A.C., Shaffer A.K., Trites A.W., Jarman S.N. (2013). Quantifying sequence proportions in a DNA-based diet study using Ion Torrent amplicon sequencing: Which counts count?. Mol. Ecol. Resour..

[53] Boessenkool S., Epp L.S., Haile J., Bellemain E., Edwards M., Coissac E., Willerslev E., Brochmann C. (2012). Blocking human contaminant DNA during PCR allows amplification of rare mammal species from sedimentary ancient DNA. Mol. Ecol..

[54] Shehzad W., Riaz T., Nawaz M.A., Miquel C., Poillot C., Shah S.A., Pompanon F., Coissac E., Taberlet P. (2012). Carnivore diet analysis based on next-generation sequencing: Application to the leopard cat (*Prionailurus bengalensis*) in Pakistan. Mol. Ecol..

[55] Takahashi M., DiBattista J.D., Jarman S., Newman S.J., Wakefield C.B., Harvey E.S., Bunce M. (2020). Partitioning of diet between species and life history stages of sympatric and cryptic snappers (Lutjanidae) based on DNA metabarcoding. Sci. Rep..

[56] Xiong F., Shu L., Zeng H., Gan X., He S., Peng Z. (2022). Methodology for fish biodiversity monitoring with environmental DNA metabarcoding: The primers, databases and bioinformatic pipelines. Water Biol. Secur..

[57] Keck F., Couton M., Altermatt F. (2023). Navigating the seven challenges of taxonomic reference databases in metabarcoding analyses. Mol. Ecol. Resour..

[58] Mychek-Londer J.G., Chaganti S.R., Heath D.D. (2020). Metabarcoding of native and invasive species in stomach contents of Great Lakes fishes. PLoS ONE.

[59] Evans H.K., Bunch A.J., Schmitt J.D., Hoogakker F.J., Carlson K.B. (2021). High-throughput sequencing outperforms traditional morphological methods in Blue Catfish diet analysis and reveals novel insights into diet ecology. Ecol. Evol..

[60] Kume G., Kobari T., Hirai J., Kuroda H., Takeda T., Ichinomiya M., Komorita T., Aita-Noguchi M., Hyodo F. (2021). Diet niche segregation of co-occurring larval stages of mesopelagic and commercially important fishes in the Osumi Strait assessed through morphological, DNA metabarcoding, and stable isotope analyses. Mar. Biol..

[61] Nalley E.M., Donahue M.J., Heenan A., Toonen R.J. (2022). Quantifying the diet diversity of herbivorous coral reef fishes using systematic review and DNA metabarcoding. Environ. DNA.

[62] Kim H., Kim J., Choi J.W., Ahn K.S., Park D.I., Kim S. (2023). A streamlined pipeline based on HmmUFOtu for microbial community profiling using 16S rRNA amplicon sequencing. Genom. Inf..

[63] Wells C.D., Paulay G., Nguyen B.N., Leray M. (2021). DNA metabarcoding provides insights into the diverse diet of a dominant suspension feeder, the giant plumose anemone *Metridium farcimen*. Environ. DNA.

[64] Kim H.G., Kwak I.-S. (2022). Determination of spatial and individual variations in the dietary composition of *Pennahia argentata* in coastal waters of South Korea using metabarcoding and morphological analyses. Reg. Stud. Mar. Sci..

[65] Li L., Yin X., Wan Q., Rusitanmu D., Han J. (2024). Diet Diversity of the Fluviatile Masu Salmon, Oncorhynchus masou (Brevoort 1856) Revealed via Gastrointestinal Environmental DNA Metabarcoding and Morphological Identification of Contents. Biology.

[66] Yao M., Zhang S., Lu Q., Chen X., Zhang S.-Y., Kong Y., Zhao J. (2022). Fishing for fish environmental DNA: Ecological applications, methodological considerations, surveying designs, and ways forward. Mol. Ecol..

[67] Lu Q., Li M., Tian Q., Jin X., Hu Y., Cai J., Fang S., Wang Y., Ding P., Thomas L. (2023). Food webs reveal coexistence mechanisms and community organization in carnivores. Curr. Biol..

[68] Jo H., Gim J.-A., Jeong K.-S., Kim H.-S., Joo G.-J. (2014). Application of dna barcoding for identification of freshwater carnivorous fish diets: Is number of prey items dependent on size class for micropterus salmoides?. Ecol. Evol..

[69] Li Y., Chen B., Bao X., Zhou Z., Liu W., Li Y. (2023). Preliminary dietary analysis of *Hyporhamphus sajori* juveniles based on DNA metabarcoding. J. Fish. Sci. China.

[70] Xu J., Wang S., Huang H., Yao X., Zhao K., Qiu B., Dai M. (2017). Research on spatial difference of diet of cultured abalone by stable carbon and nitrogen isotope. J. Fuzhou Univ. (Nat. Sci. Ed.).

[71] Tófoli R.M., Alves G.H.Z., Higuti J., Cunico A.M., Hahn N.S. (2013). Diet and feeding selectivity of a benthivorous fish in streams: Responses to the effects of urbanization. J. Fish Biol..

[72] Alves V.E.N., Patrício J., Dolbeth M., Pessanha A., Palma A.R.T., Dantas E.W., Vendel A.L. (2016). Do different degrees of human activity affect the diet of Brazilian silverside *Atherinella brasiliensis*?. J. Fish Biol..

[73] Harms-Tuohy C.A., Schizas N.V., Appeldoorn R.S. (2016). Use of DNA metabarcoding for stomach content analysis in the invasive lionfish Pterois volitans in Puerto Rico. Mar. Ecol. Prog..

[74] Pompanon F., Deagle B.E., Symondson W.O., Brown D.S., Jarman S.N., Taberlet P. (2012). Who is eating what: Diet assessment using next generation sequencing. Mol. Ecol..

[75] Deagle B.E., Thomas A.C., McInnes J.C., Clarke L.J., Vesterinen E.J., Clare E.L., Kartzinel T.R., Eveson J.P. (2019). Counting with DNA in metabarcoding studies: How should we convert sequence reads to dietary data?. Mol. Ecol..

[76] Liu G., Ning Y., Xia X.F., Gong M.H. (2018). The application of high-throughput sequencing technologies to wildlife diet analysis. Acta Ecol. Sin..

